# Editorial: Macromolecular interactions in signaling pathways: from classical approaches to virtual reality

**DOI:** 10.3389/fendo.2024.1388849

**Published:** 2024-02-28

**Authors:** Marcello Leopoldo, Marialessandra Contino, Stuart Maudsley, Milka Vrecl

**Affiliations:** ^1^ Department of Pharmacy - Drug Sciences, University of Bari Aldo Moro, Bari, Italy; ^2^ Receptor Biology Lab, University of Antwerp, Antwerp, Belgium; ^3^ Veterinary Faculty, Institute of Preclinical Sciences, University of Ljubljana, Ljubljana, Slovenia

**Keywords:** GPCRs, signal transduction, protein-protein interactions, dimerization, internalization

This Research Topic aimed to provide an overview of methodological approaches to the study of protein-protein interactions (PPIs) and to shed light on the complex architecture of the transmembrane receptor signaling interactome that enables the propagation, amplification, and modulation of signals that ultimately lead to cellular responses. The focus was on advances in biophysical methods for monitoring PPIs in living cells, receptor oligomerization, bioinformatics, and computational tools for predicting PPIs, as well as experimental challenges and future trends in PPI research. A selection of review and original research articles was compiled focusing on homo-/heteromerization of G protein-coupled receptors (GPCRs) (Dale et al.; Nemoto et al.; Treppiedi et al.) and methodological challenges in the study of constitutively active orphan GPCRs (Mavri et al.). The review article by Dale et al. discusses the importance of GPCR heteromerization for receptor pharmacology and physiology, the methodological approaches for monitoring heteromer formation/identification and the criteria that a heteromer must meet to be recognized as physiologically relevant. In addition, methods for validation of heteromers are presented to detect: *i*) proximity of receptor promoters, *ii*) distinct biochemical properties of heteromers, and *iii*) disruption of heteromer function. The study by Nemoto et al. then reports on the development of a user-friendly and freely accessible GPCR–GPCR interacting pair predictor (GGIP) web server (https://protein.b.dendai.ac.jp/GGIP/). The article also discusses examples of applications for GGIP, with a focus on analyzing disease-associated mutations that potentially affect GPCR–GPCR interactions. The third article dealing with homo-/heteromerization of GPCRs is from Treppiedi et al. and examines the existence and physiological significance of homo- and heteromerization of somatostatin receptors (SSTs) in the pituitary gland, including the role of the SST_2_- and SST_5_-binding partner, i.e. the actin-binding protein filamin A (FLNA), in this process. They employed the proximity ligation assay for *in situ* visualization and quantification of SST_2_/SST_5_ dimerization in rat pituitary GH-secreting (GH3) cells and in human melanoma cells, A7 (FLNA-expressing) and M2 (FLNA-deficient). In all three cell lines, SST_2_/SST_5_ homo- and heteromerization was observed, with FLNA regulating the formation of heterodimers and the SST_2_ and SST_5_ agonist-induced intracellular trafficking.

Finally, the original article by Mavri et al. reports a comprehensive characterization of BILF1 encoded by human Epstein-Barr virus (EBV) and BILF1 receptors encoded by three porcine lymphotropic herpesviruses (PLHV1-3). Using different cell lines (human embryonic kidney 293 (HEK-293), porcine kidney 15 (PK-15) and CRISPR/Cas9-modified HEK-293A pan-knockout cells) and experimental *in vitro* approaches, PLHVs were characterized with respect to their localization, constitutive signaling and internalization as well as their ability to downregulate MHC-I. PLHV1 was also detected in lymphatic tissue from pigs with post-transplant lymphoproliferative disease (PTLD), providing initial evidence that pigs infected with PLHV1 could serve as an *in vivo* model for the study of PTLD.

Taken together, it can be summarized that proximity-based biophysical methods continue to be extensively employed in PPI research. In addition, there is a burgeoning trend towards utilizing computational methods for predicting interacting partners as well as different cell-based assays to validate potential interaction partners in an appropriate physiological environment. Unfortunately, integration of *in vitro, in cellula* and *in vivo* models along with AI (artificial intelligence) was not sufficiently addressed in this Research Topic.

## Author contributions

ML: Writing – review & editing. MC: Writing – review & editing. SM: Writing – review & editing. MV: Writing – original draft, Writing – review & editing.

